# Toward a hybrid exoskeleton for crouch gait in children with cerebral palsy: neuromuscular electrical stimulation for improved knee extension

**DOI:** 10.1186/s12984-020-00738-7

**Published:** 2020-09-03

**Authors:** Blynn L. Shideler, Thomas C. Bulea, Ji Chen, Christopher J. Stanley, Andrew J. Gravunder, Diane L. Damiano

**Affiliations:** National Institutes of Health, Clinical Center, Rehabilitation Medicine Department, Functional & Applied Biomechanics Section, Bldg 10 CRC Rm 1-1469, 10 Center Dr. MSC 1604, Bethesda, MD 20892-1604 USA

**Keywords:** Functional electrical stimulation (FES), Exoskeleton, Crouch gait, Graphical user interface (GUI)

## Abstract

**Background:**

Neuromuscular Electrical Stimulation (NMES) has been utilized for many years in cerebral palsy (CP) with limited success despite its inherent potential for improving muscle size and/or strength, inhibiting or reducing spasticity, and enhancing motor performance during functional activities such as gait. While surface NMES has been shown to successfully improve foot drop in CP and stroke, correction of more complex gait abnormalities in CP such as flexed knee (crouch) gait remains challenging due to the level of stimulation needed for the quadriceps muscles that must be balanced with patient tolerability and the ability to deliver NMES assistance at precise times within a gait cycle.

**Methods:**

This paper outlines the design and evaluation of a custom, noninvasive NMES system that can trigger and adjust electrical stimulation in real-time. Further, this study demonstrates feasibility of one possible application for this digitally-controlled NMES system as a component of a pediatric robotic exoskeleton to provide on-demand stimulation to leg muscles within specific phases of the gait cycle for those with CP and other neurological disorders who still have lower limb sensation and volitional control. A graphical user interface was developed to digitally set stimulation parameters (amplitude, pulse width, and frequency), timing, and intensity during walking. Benchtop testing characterized system delay and power output. System performance was investigated during a single session that consisted of four overground walking conditions in a 15-year-old male with bilateral spastic CP, GMFCS Level III: (1) his current Ankle-Foot Orthosis (AFO); (2) unassisted Exoskeleton; (3) NMES of the *vastus lateralis*; and (4) NMES of the *vastus lateralis* and *rectus femoris*. We hypothesized in this participant with crouch gait that NMES triggered with low latency to knee extensor muscles during stance would have a modest but positive effect on knee extension during stance.

**Results:**

The system delivers four channels of NMES with average delays of 16.5 ± 13.5 ms. Walking results show NMES to the *vastus lateralis* and *rectus femoris* during stance immediately improved mean peak knee extension during mid-stance (*p* = 0.003*) and total knee excursion (*p* = 0.009*) in the more affected leg. The electrical design, microcontroller software and graphical user interface developed here are included as open source material to facilitate additional research into digitally-controlled surface stimulation (github.com/NIHFAB/NMES).

**Conclusions:**

The custom, digitally-controlled NMES system can reliably trigger electrical stimulation with low latency. Precisely timed delivery of electrical stimulation to the quadriceps is a promising treatment for crouch. Our ultimate goal is to synchronize NMES with robotic knee extension assistance to create a hybrid NMES-exoskeleton device for gait rehabilitation in children with flexed knee gait from CP as well as from other pediatric disorders.

**Trial registration:**

clinicaltrials.gov, ID: NCT01961557. Registered 11 October 2013; Last Updated 27 January 2020.

## Background

Cerebral palsy (CP) is the most common child onset neuromotor disability, affecting over 17 million people worldwide [1]. CP is caused by a brain injury during early development that leads to motor deficits, particularly involving gait and posture [2]. Crouch gait, the most prevalent and debilitating gait disorder in CP, is characterized by excessive knee flexion in early and/or mid stance [3]. Muscle weakness, spasticity, contractures, and impaired selective motor control have all been shown to contribute to crouch gait in CP to varying degrees across individuals [4]. Crouch increases the energy demands of walking [5] and often progresses with age and growth, despite treatment, leading to mobility decline starting as early as adolescence [6].

Current treatment of crouch gait includes both invasive and non-invasive options [4]. Orthopaedic surgeries [7, 8], such as hamstring lengthening [4], are advocated when hamstring muscles become excessively short; however, repeat lengthenings are contraindicated. Botulinum toxin injections to spastic muscles [9], and physical therapy approaches such as strengthening lower limb extensors have also been utilized [10, 11] for crouch gait. These may demonstrate some limited improvements in the short term, but long-term deficits frequently persist regardless of treatment [12, 13]. Wearable lower limb exoskeletons provide a new avenue for gait rehabilitation [14–19]. A pediatric robotic exoskeleton recently developed at the National Institutes of Health Clinical Center designed specifically to improve crouch gait in children with CP was shown to improve knee extension during stance without decreasing muscle activation in the extensor muscles throughout the gait cycle after only a short accommodation period to walking with the device in the laboratory [14, 15, 20]. These results demonstrate a promising possibility for exoskeletons to be used as a training tool because they improve crouch gait kinematics in children with CP while still maintaining and even potentially augmenting volitional muscle control. We propose that incorporation of NMES with motorized assistance is integral to achieving optimal long-term outcomes for those with crouch gait. NMES has the unique capability to increase muscle strength and size [21], inhibit or reduce spasticity [22, 23], and enhance motor performance during specific tasks through precisely timed muscle activation or sensory cues [24]. Furthermore, peripheral muscle stimulation may stimulate greater neuroplastic changes in sensorimotor brain regions during gait training [25].

Muscle activation, either voluntary or stimulated, is important for maintaining strength. Progressive resistance training, which uses higher loads than normally encountered in everyday life, has been shown to reliably increase strength in targeted muscles and may improve functional motor outcomes in children with CP [26]. Apart from traditional strength training, electrical stimulation has also been shown to improve muscle strength and increase muscle size [27]. Functional electrical stimulation (FES) is a term describing the use of electrical stimulation specifically for supplementing or replacing function lost in individuals with neurological impairments [28]. A review by Seifart et al. explained that “FES can be distinguished from NMES in that NMES is aimed at muscle strengthening and/or spasticity reduction and is not always applied functionally,” and charged researchers to “clearly distinguish FES from NMES in future studies.” [29] Because our goals in CP extend beyond the direct functional effects, we chose to use the broader term neuromuscular electrical stimulation (NMES) here, with the caveat that we are only reporting its immediate effect on lower limb kinematics during gait in this first participant with CP.

NMES and FES have been used in children with CP to improve foot drop during swing phase of gait with evidence of increased muscle size on ultrasound after months of using the device for nearly 6 h per day on average [30, 31]. Khamis et al. were the first group to report the use of FES to address crouch in an 18 year old individual with CP by applying FES to the quadriceps muscles during periods within the stance phase when the knee was supposed to be extending, as detected by a force-sensitive gait sensor placed beneath the patient’s heel [32]. After months of in-home training with the device, gait speed increased and knee extension minimally improved during FES use. The level of quadriceps activation by FES was not reported, and changes in muscle strength and size were not examined before and after training; therefore, the role of FES versus walking practice in producing the gait improvement remains unclear [32].

We postulate that quadriceps NMES precisely synchronized with volitional knee extension during gait will augment knee extension in those with crouch. In the immediate or short-term, we propose that NMES will provide an orthotic effect to improve knee biomechanics during gait and may add some additional antigravity support to make walking easier. Several groups have begun to investigate the combination of exoskeleton assistance and simultaneous lower-limb FES in healthy participants [33, 34] and adults with complete spinal cord injury (SCI) [35–39]. Delivering FES intermittently throughout the gait cycle with real-time control can reduce the extent of muscle fatigue while receiving stimulation [33, 35]. This paper describes a method of calibrating and controlling a surface NMES device with a digital microcontroller and synchronizing NMES with the gait cycle using a finite state machine implemented as part of a pediatric robotic exoskeleton. Thus, the aims of this study are to: (1) outline the design of a custom NMES system that can trigger on-demand electrical stimulation in real-time; and (2) demonstrate the feasibility of one possible application for this NMES system as a component of a robotic exoskeleton by investigating its effects on a single participant with CP within a single session. Our ultimate goal, in the longer term, is to demonstrate that NMES-assisted gait training paradigms will provide targeted muscle strengthening during gait training; strengthen motor pathways and facilitate motor learning by providing a cutaneous sensory timing cue to activate muscles; and additionally result in reciprocal inhibition of potentially spastic and/ or co-activating antagonist muscles.

## Methods

### Digitally-controlled neuromuscular electrical stimulation

**Fig. 1:**
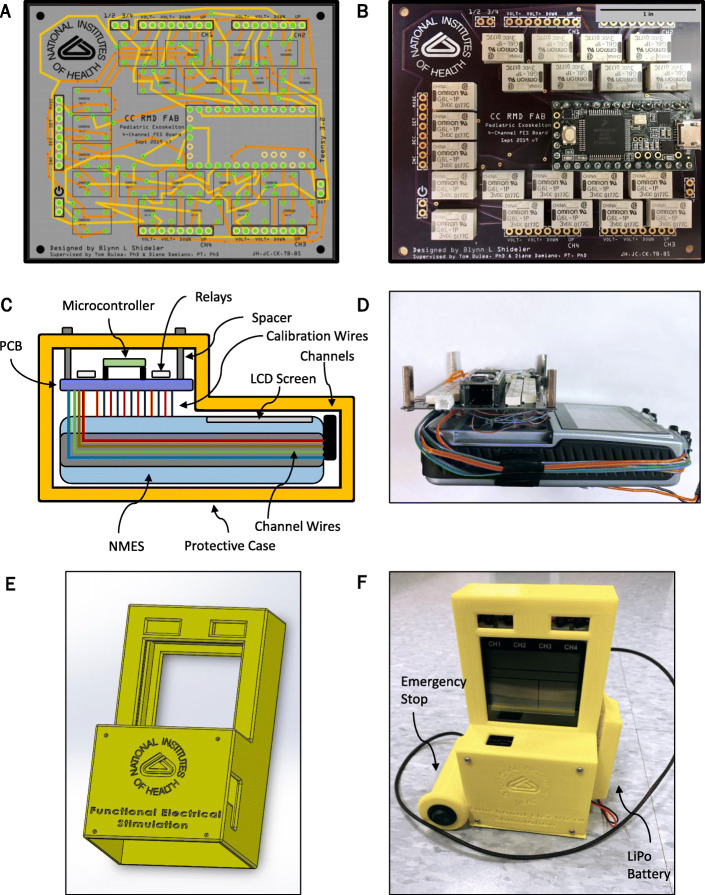
The custom-designed digitally-controlled surface NMES system. **a** custom printed circuit board (PCB) wiring schematic and **b** printed board that houses a Teensy 3.2 microcontroller, a system of 21 Omron Electronic Components G6L-1P-DC3 SPST-NO electromechanical relays, and inputs for a LiPo battery and external digital input signals, with scale bar for reference. **c** schematic of the NMES system and **d** wired system prototype. **e** a 3D model of the custom protective case and **f** the enclosed system used for this study

In order to deliver surface NMES within discrete phases of the gait cycle, it was necessary to design an NMES controller that could be digitally calibrated and operated. While some groups have designed their own electrical stimulation units in order to control them with microprocessors [40, 41], our digital NMES system is based on the architecture of a Food & Drug Administration (FDA)-approved four-channel, eight-electrode LG-8TM Elite Electrical Muscle Stimulator (LG Med Supply). The circuits on the LG-8TM that close each of the 13 buttons for setting the electrical stimulation amplitude, pulse-width, and frequency, as well as the circuits that deliver stimulation output to each of the four channels, were replaced with a custom system of 21 relays—13 for each of the calibration operators and 8 for the four channels (2 per channel as each channel has a positive voltage output and a negative voltage output). A G6L-1P-DC3 Single Pole Single Throw (SPST) Normally-Open (NO) electromechanical relay (Omron Electronic Components) was chosen for our design due to its small size and fast, reliable switching with audible feedback. A printed circuit board (PCB) was designed using Fritzing Version 0.9.3b to house the system of 21 relays and the microcontroller (Fig. 1a, Fig. 1b).

A Teensy 3.2, containing a 32-bit ARM Cortex-M4 microcontroller, was utilized to control the relay system for calibration and stimulation delivery. The microcontroller was programmed with an adapted version of the Arduino Integrated Development Environment (IDE) (Arduino IDE 1.8.8 + Teensyduino 1.46). Twenty-one digital output pins on the Teensy 3.2 were wired to send a 3.3 V signal to close a corresponding electromechanical relay on the custom PCB, which in turn digitally presses the button associated with that relay on the LG-8TM Elite four-channel NMES (Fig. 1c and d). The Teensy 3.2 received commands by constantly searching for input from the serial monitor, with specific integer inputs mapped to each electromechanical relay trigger.

A custom case was designed using SolidWorks Professional 2018 × 64 Edition (Dassault Systems) and manufactured with a MakerBot 5th Generation Replicator 3D Printer (MakerBot Industries) to enclose the system of wiring between the LG-8TM Elite four-channel, eight-electrode NMES device by LG Med Supply and the custom printed circuit board relay system, (Fig. 1e, Fig. 1f). In this infrastructure, original liquid crystal display (LCD) screen function of the LG Med Supply NMES device was maintained. Additionally, an emergency stop switch was hardwired into the main power supply of the NMES to immediately power off the NMES via a mechanical switch held by the user or therapist (Fig. 1).

**Fig. 2:**
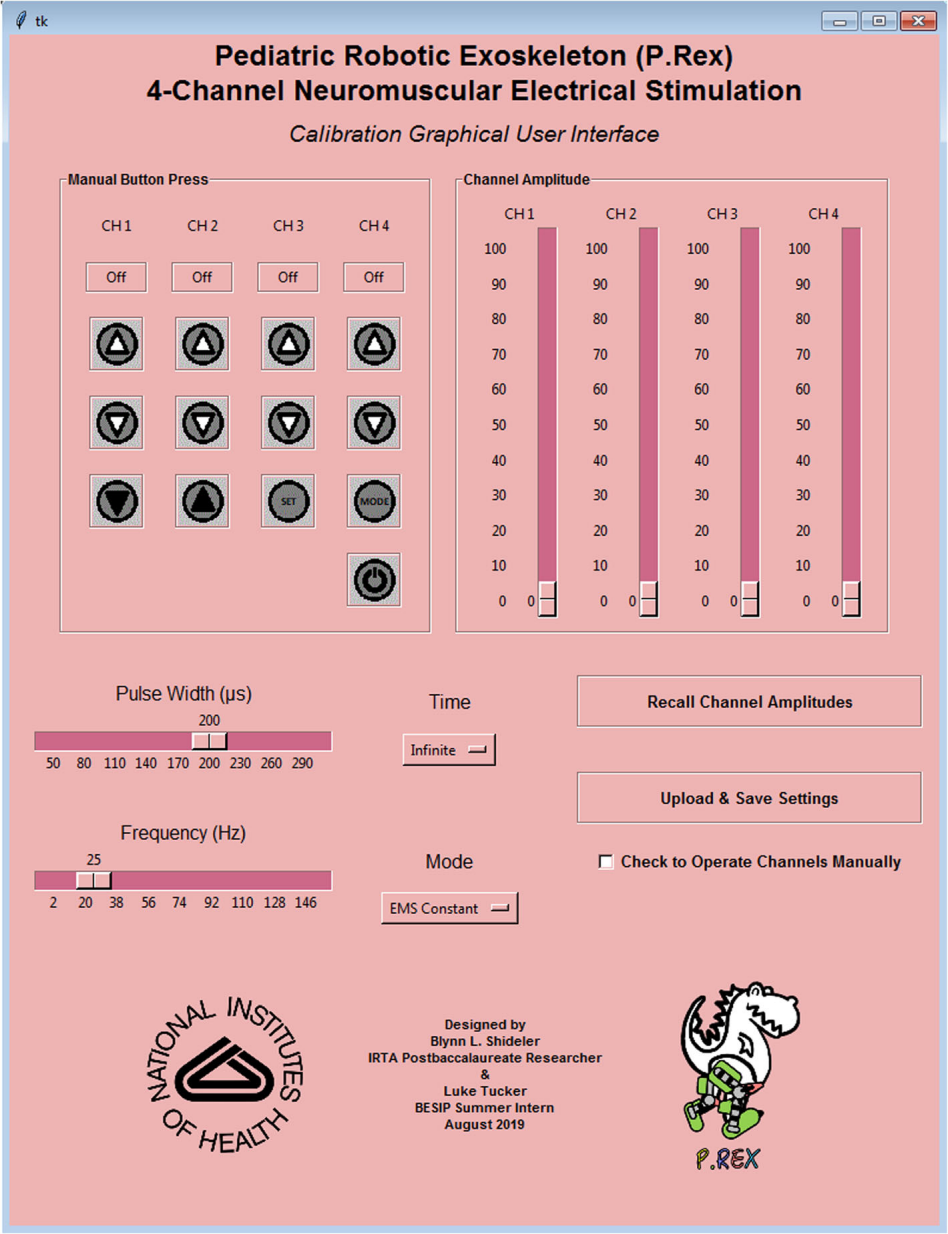
A graphical user interface (GUI) to communicate with the Teensy 3.2 microcontroller operating the neuromuscular electrical stimulator for digital calibration and control of the neuromuscular electrical stimulation

**Fig. 3:**
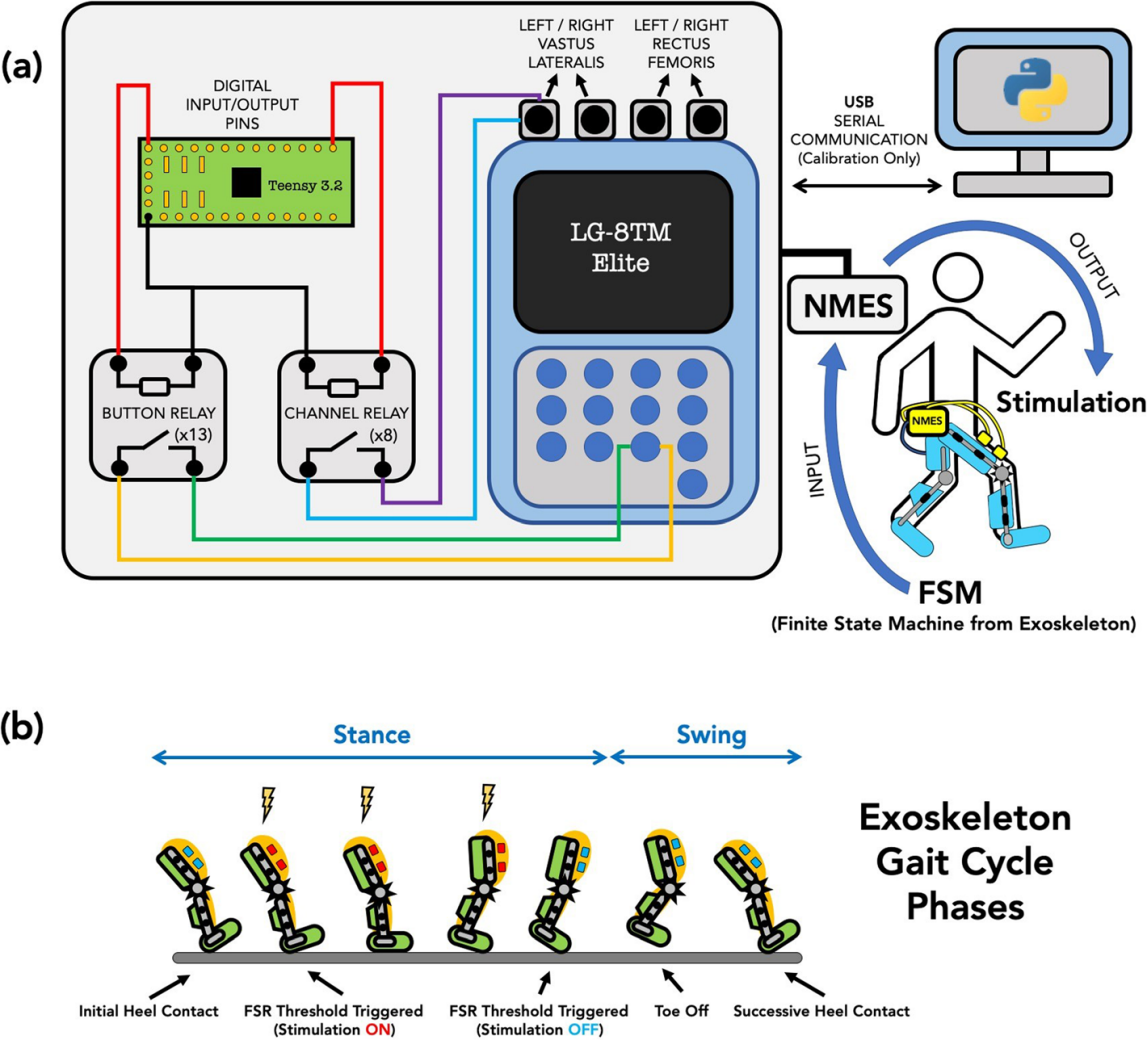
**a** Schematic of the synchronized neuromuscular electrical stimulation (NMES) and exoskeleton communication. The NMES device is calibrated under complete digital control from a Teensy 3.2 microcontroller and system of electromechanical relays housed on a printed circuit board (PCB). Front-end calibration occurs on a Python desktop graphical user interface. The NMES synchronizes with the exoskeleton motor control via digital input from the exoskeleton’s finite state machine. **b** Schematic of the gait cycle, showing how the timing of stimulation delivery during stance phase as determined by the Finite State Machine (FSM) deviates slightly from the traditional biomechanical definition

Finally, a graphical user interface (GUI) was designed using Tkinter Graphical User Interface package on Python 3.7.4 (Python Software Foundation) to calibrate the NMES waveforms by setting the stimulation amplitude, frequency, and pulse-width. The GUI also allows a user to activate and de-activate the NMES channels (Fig. 2). All code and files necessary to operate the GUI are available open source (github.com/NIHFAB/NMES). The schematic flow of communication between the graphical user interface, the NMES calibration and synchronization system, and input from the pediatric robotic exoskeleton’s FSM is illustrated in Fig. 3.

### Pediatric robotic exoskeleton

**Fig. 4:**
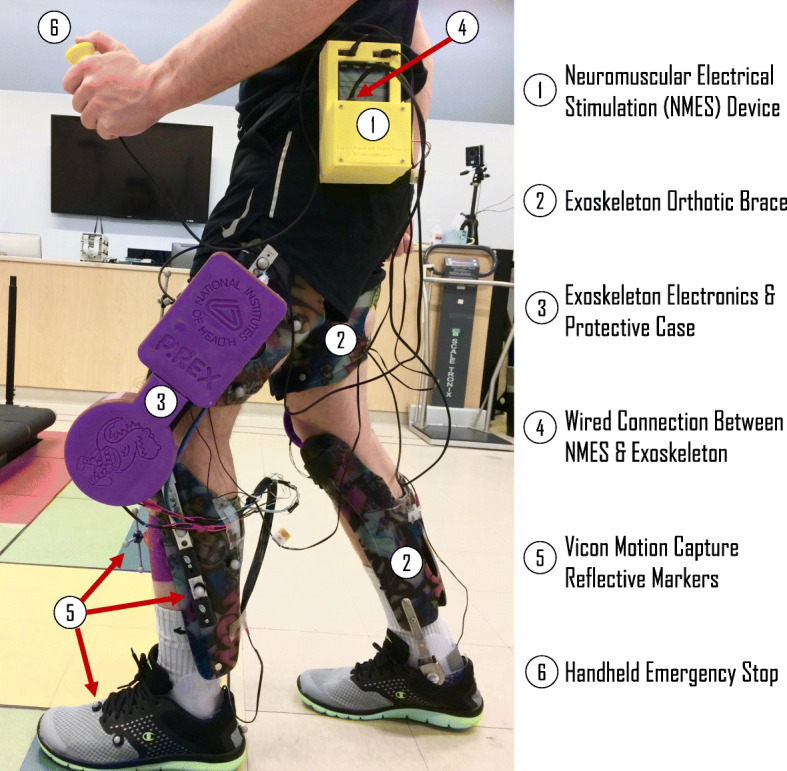
Representation of the experimental setup for clinical testing of walking in the pediatric robotic exoskeleton with synchronized NMES across the Functional & Applied Biomechanics Laboratory in the National Institutes of Health Clinical Center

The NIH pediatric robotic exoskeleton utilized its incorporated sensors to identify the gait cycle phases in real-time. The NMES was triggered by the exoskeleton controller to deliver stimulation to the quadriceps muscles throughout stance phase, i.e., when the FSR value was above the tuned threshold indicating foot contact with the ground. The NIH pediatric exoskeleton is a lightweight (3.2 kg), modular device based on the architecture of a knee-ankle-foot orthosis [14, 15, 20] designed to provide motorized assistance at the knee joint to augment knee extension during walking, and thus is specifically designed for rehabilitation of crouch gait in children with CP [14, 42]. In this study, however, the motors provided only enough torque to compensate for the friction and inertia of the exoskeleton and thus were not actively assisting knee extension. Both legs of the exoskeleton are equipped with a combination of sensors including a quadrature encoder (Maxon Motor), a torque sensor (Transducer Techniques), and a force sensitive resistor (Interlink Electronics) to track knee angle and angular velocity, knee joint torque, and foot-ground contact, respectively [20]. A Teensy 3.2 microcontroller (PJRC Electronic Projects) is programmed to read each leg’s exoskeleton sensor data to construct a finite state machine (FSM) and identify the user’s distinct gait cycle state in each limb in real-time. The hybrid pediatric robotic NMES-exoskeleton is shown in Fig. 4.

### Benchtop testing synchronization experimental setup

To evaluate the design of the digitally-controlled NMES system and validate its ability to deliver stimulation in response to state changes in the FSM of the pediatric robotic leg exoskeleton, the system’s FSM and the NMES voltage output were measured over 20 state change (swing➔stance) simulations. State changes were simulated by manually applying a force to the FSR of the exoskeleton above a 1 N threshold in 1-s intervals, which triggers a change from the swing to stance phase in the FSM. The FSM output was collected from the embedded software of the exoskeleton, sampled at 80 Hz, and the NMES output was collected using a digital oscilloscope (TDS 2024B, Tektronix), sampled at 2500 Hz. The two data streams were synchronized using a square pulse trigger to align the data sets in post-processing. To simulate its use during walking, the NMES system was programmed to trigger electrical stimulation during stance phase and to turn off during swing phase. FSM state change and NMES signals were analyzed in post-processing using MATLAB 2018b (MathWorks). The time points when the signals were determined to reach the on-state were measured and recorded. On-state of the NMES was defined as the moment where a 24 V voltage value (i.e., the programmed stimulation amplitude) was achieved. Onset delay was calculated as the average time difference between state-change of the FSM and on-state of NMES for each of the 20 simulated state changes.

### Clinical investigation: a 15-year-old male with CP

A 15-year-old male with bilateral spastic CP (Height = 162 cm; Weight = 39.9 kg) completed a single session evaluation of the NMES-assisted pediatric robotic exoskeleton. This study was performed as part of a larger protocol evaluating the effects of robotic exoskeletons on gait in children with CP (Protocol #13-CC-0210). After informed consent into our IRB-approved protocol, several clinical assessments were performed by a physician during an initial visit, including the Modified Ashworth Scale (MAS) and Gross Motor Function Classification System (GMFCS) assessments. The participant showed mild spasticity (MAS = 1 or 1+) in the right rectus femoris, and bilateral hamstrings, vastus lateralis, and gastrocnemius muscles. The participant was classified as GMFCS level III, and he required use of forearm crutches to walk. An initial gait analysis assessment in his typical walking condition—which included wearing AFOs and use of forearm crutches—and measurements for fabrication of a custom exoskeleton were also performed. The second visit of the protocol included initial exoskeleton fitting and tuning of the control system, including the FSM. The exoskeleton fitting included adjusting the ankle of the exoskeleton to match the range of motion allowed by the participant’s own AFOs. The second visit, and all subsequent visits, also included calibration of the NMES parameters. NMES electrode pads were placed on the skin overlying the center point of the widest portion of the *vastus lateralis* and *rectus femoris* muscles on each limb. Calibration was performed systematically on each muscle of each leg by first increasing the amplitude by the minimum resolution of the stimulator until a visible muscle contraction was induced, then increasing the frequency by the minimum resolution until the participant perceived a continuous stimulation with minimal or no discomfort, then finally increasing the pulse width by the minimum resolution to the maximum tolerable level. The minimum resolution of the stimulator (LG-8TM Elite, LG Med Supply) was 0.8 mA for amplitude, 1 Hz for frequency, and 10 μs for pulse width. Stimulation parameters were implemented at their calibrated values for all NMES walking conditions (Left and Right *Vastus Lateralis*: Amplitude = 36 mA; Frequency = 25 Hz; Pulse Width = 200 μs // Left and Right *Rectus Femoris*: Amplitude = 44 mA; Frequency = 25 Hz; Pulse Width = 200 μs). Isolated stimulation of the *vastus lateralis* was investigated as a walking condition because this muscle spans a single joint and was easily accessible for electrode placement. Another reason was that the *rectus femoris*, while a strong knee extensor, also provides flexion at the hip joint which may be counterproductive in some patients. However, we wanted to pilot this muscle as well in this patient to see if its addition improved or worsened his knee extension and also to demonstrate the capability of our NMES device to simultaneously activate multiple muscles. The participant completed 5 visits to practice walking with the exoskeleton and NMES systems. On the 6th visit, gait analysis was performed to assess the effect of the NMES system on walking. Reflective markers were placed on the pelvis and lower extremities for 3D motion capture (Vicon Motion Systems). The participant walked 15 m lengths across the laboratory. The participant walked with forearm crutches with a physical therapist walking behind him and holding the hybrid NMES-exoskeleton handheld emergency stop. A passive body harness for safety was worn but did not provide body-weight support (Aretech ZeroG).

The participant walked for approximately 10 min in each of four conditions: (1) walking in the participant’s prescribed ankle-foot orthosis (AFO), (2) baseline walking with the exoskeleton in zero-torque mode with no NMES (“Exoskeleton”), (3) walking in the exoskeleton in zero-torque mode with NMES to the *vastus lateralis* only (“NMES: VL”), and (4) walking in the exoskeleton in zero-torque mode with NMES to the *vastus lateralis* and *rectus femoris* (“NMES: VL + RF”). Zero-torque mode of the exoskeleton is designed to be transparent to the wearer by compensating for inertial effects, as described previously [20]. Exoskeleton ankle range of motion, adjusted to match the participant’s own AFO, was the same for all trials. The exoskeleton FSM was programmed to split the gait cycle into two distinct states: stance phase and swing phase. The NMES was programmed to apply stimulation to the designated muscles during stance phase only (Fig. 3b). Kinematic and spatiotemporal gait parameters were calculated for all conditions and analyzed using Visual3D (C-Motion). The participant was seated for rest periods up to 10 min in between walking conditions to minimize any effect of fatigue on walking. For biomechanical analysis, walking was split into gait cycles for each limb spanning from heel strike to heel strike. Stance and swing were also determined from motion capture using heel strike and toe-off events. Note that these phases differ from the on/off initiation of NMES from the exoskeleton FSM, which occurred when the FSR under the foot exceeded (and fell below) a pre-calibrated threshold, respectively. It was therefore anticipated that NMES would be delivered during a subset of the stance phase determined by motion capture (Fig. 3b). Thus, the period of NMES was calculated offline to determine the exact portion of stance that NMES was administered to the quadriceps. Primary outcome measures were gait speed and knee angle kinematics, particularly peak knee extension during stance and total knee angle excursion.

### Statistical analysis

Repeated-measures, one-factor ANOVA procedures were performed at the α = 0.05 significance level, to compare differences in peak knee extension during stance, total knee excursion, and gait speed between the three experimental conditions—(1) baseline exoskeleton walking, (2) exoskeleton walking with synchronized NMES to only the *vastus lateralis* and (3) exoskeleton walking trials with synchronized NMES to both the *vastus lateralis* and the *rectus femoris*—while controlling for differences in stride number, to isolate the effect of the NMES conditions. Post-hoc pairwise comparisons were made using the Bonferroni method for multiple comparisons correction. Statistical analysis was performed using MATLAB 2018b (MathWorks). The participant’s AFO walking condition was included here to demonstrate the effects that wearing an exoskeleton had on his gait speed and posture and was only compared to the zero-torque exoskeleton condition using paired t-tests (*p* < 0.05).

## Results

Initial testing of the proposed system verified that the microprocessor control system could operate each of the 13 calibration relays as well as each of the four stimulation output channels completely and independently. Further, this initial testing confirmed that communication could successfully and reliably be transmitted and received between the digital control of the NMES system and the FSM of the exoskeleton. Each stimulation channel outputs 0-80 mA, depending on the amplitude of the waveform delivered; thus, at maximum output on all four channels, the 2000mAh LiPo battery used to power this custom, digitally-controlled NMES device could operate the system for about 6 h on a single charge.

**Fig. 5:**
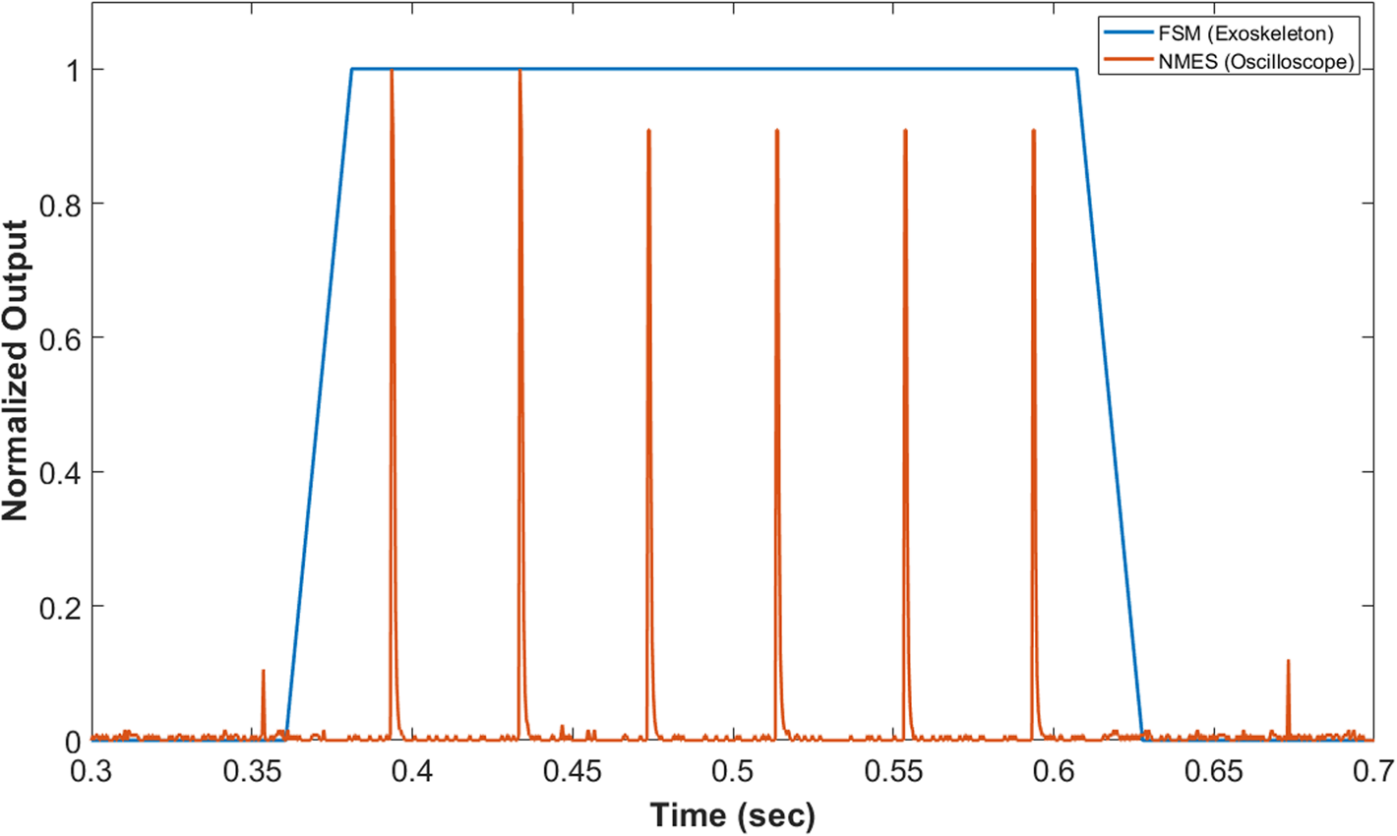
Benchtop testing neuromuscular electrical stimulation (NMES) output synchronized with a state change detected by the finite state machine (FSM) of the pediatric robotic exoskeleton; one simulated state change with aligned signals from the NMES and FSM shown for example. Normalized output = 1 indicates stance phase on the FSM and maximum voltage from the NMES

The average delay between a state change in the exoskeleton FSM and the delivery of NMES output over 20 simulated state transitions was 16.6 ± 13.5 ms. Figure 5 shows representative example of NMES output synchronized with an FSM state change command.

**Fig. 6:**
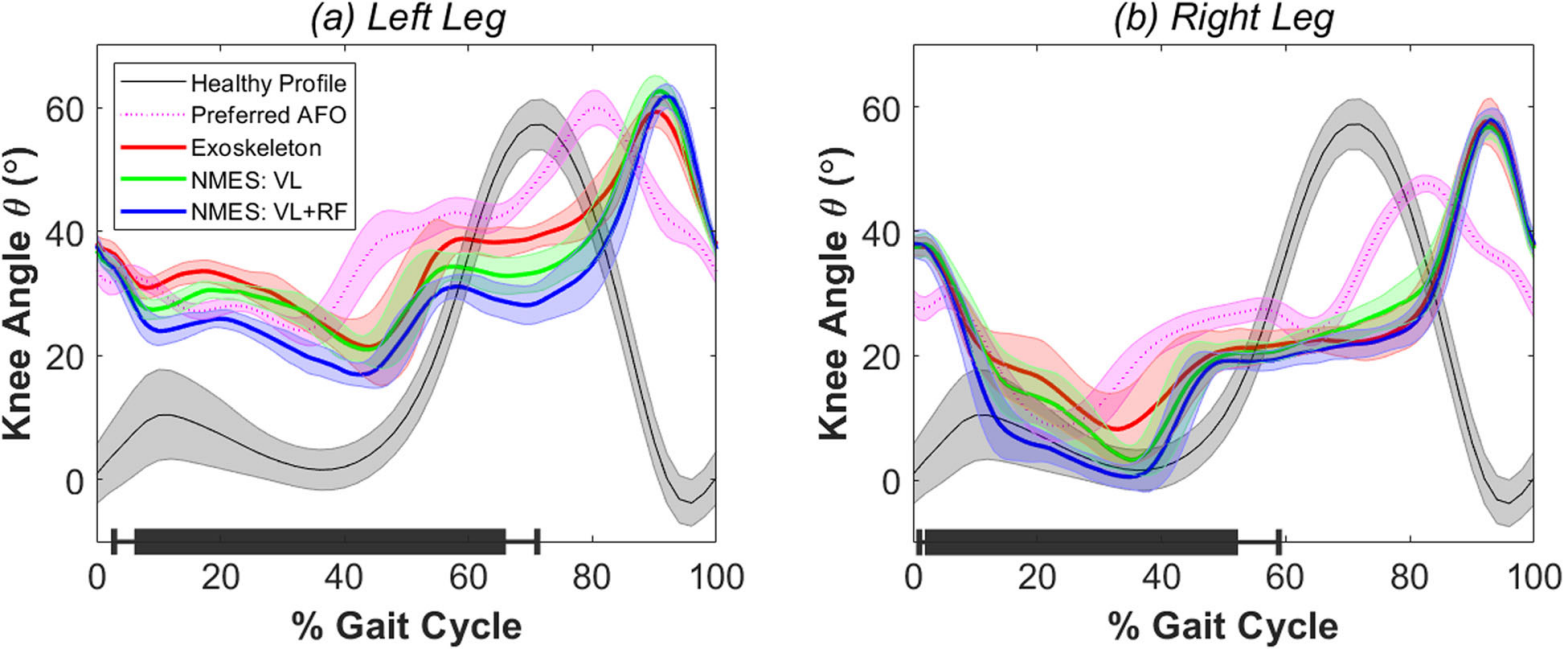
Knee angle profiles averaged for each gait cycle during all four walking conditions in **a** left leg and **b** right leg. Average profile over all gait cycles shown in bold for each condition with transparent spread representing ±1 standard deviation. Black bars along horizontal axes show the average gait cycle duration ±1 standard deviation when electrical stimulation was administered in each limb. The average knee angle profile of a healthy gait pattern from a previous study [20] is shown in grey for reference

Kinematic knee angle measures during baseline exoskeleton walking and exoskeleton walking with synchronized NMES conditions in each limb are shown in Fig. 6. Peak knee extension angle during stance showed generally more crouch in the left knee (Fig. 6, Table 1). A significant difference was found between exoskeleton walking with and without NMES in peak knee extension angle in the left leg (*p* = 0.003*) but not the right leg (*p* = 0.064). Post-hoc analysis revealed synchronized NMES to the *vastus lateralis* and *rectus femoris* significantly improved peak knee extension during mid-stance from 18.8° ± 2.5° to 15.9° ± 1.7° in the left leg compared to baseline exoskeleton walking (*p* < 0.001*). Administering NMES to only the *vastus lateralis* resulted in no significant improvement from baseline exoskeleton walking in peak knee extension angle during stance in the left leg, which averaged 19.5° ± 2.4° at full extension (*p* = 0.58). While the repeated-measures linear model showed no significant difference in peak right knee extension angle during stance between walking conditions, the descriptive statistics show a general trend that administering NMES to the quadriceps muscles decreases knee flexion during stance and increases total knee excursion (Fig. 6, Table 1). Similarly, total knee excursion differed significantly between exoskeleton walking with and without NMES in the left leg (*p* = 0.009*) but not the right leg (*p* = 0.217). In the left leg, post-hoc analysis showed mean total knee excursion significantly improved from 41.2° ± 3.9° in baseline exoskeleton walking to 46.4° ± 2.5° when NMES was administered to the *vastus lateralis* and *rectus femoris.* Gait speed did not differ significantly between all three exoskeleton conditions (*p* = 0.390). Comparing the prescribed AFO walking to baseline exoskeleton walking, the participant’s gait speed (*p* < 0.001*), peak knee extension angle during stance in the right (*p* = 0.023*) and left (*p* < 0.001*) knee, and mean total excursion in right (*p* < 0.001*) and left (*p* = 0.011*) knee all differed significantly. Table 1 summarizes the primary outcome measures and statistical analysis.

**Table 1:**
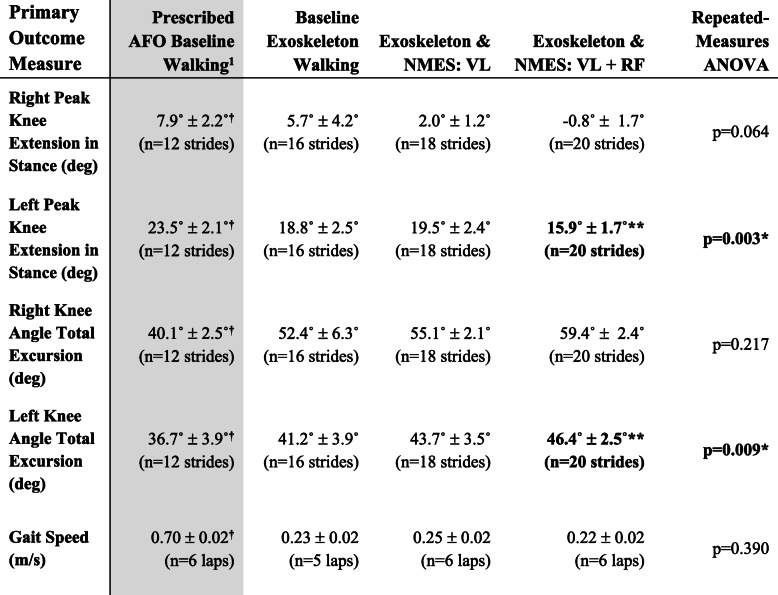
Primary outcome measures

*Abbreviations*—*VL Vastus Lateralis*, *RF Rectus Femoris*

^1^Grey shading shows prescribed AFO condition that was not included in repeated-measures ANOVA

^†^Significant difference (*p* < 0.05) between prescribed AFO baseline and baseline exoskeleton (zero assistance) walking assessed by paired t-tests

^*^Significant effect of condition at the α = 0.05 level assessed by repeated measures ANOVA

^**^Significant difference from baseline exoskeleton in post-hoc analysis at the α = 0.05 level with Bonferonni correction

Because stance phase defined by the exoskeleton FSM was determined by an FSR placed underneath the participant’s foot, NMES was always activated slightly after heel contact and prior to toe-off. The delay between first foot contact and activation of NMES averaged 0.21 ± 0.11 s and 0.06 ± 0.03 s for heel strike in the left and right limb, respectively. Similarly, NMES was turned off when the foot was unloaded but prior to toe-off by 0.66 ± 0.17 s and 1.12 ± 0.22 s for the left and right limbs, respectively.

## Discussion

This study outlines the electromechanical design, bench-top characterization, and initial clinical validation of a four-channel NMES system that can reliably trigger electrical stimulation via an external digital input signal, such as that from the FSM of the pediatric robotic exoskeleton used in this experiment. Using this system, NMES can be triggered with sufficiently low latency (16.5 ms) to ensure synchronization with gait cycle phases, e.g., stance and swing. The design of the embedded electronics, list of materials, and the Python GUI software are included as open-source material (github.com/NIHFAB/NMES) to facilitate its use by other researchers. The initial clinical testing in this study showed that the NMES system performed as intended; the participant was able to tolerate the stimulation and to maintain walking at the same speed as using the exoskeleton without stimulation. Synchronized electrical stimulation delivered to the *vastus lateralis* and/or the *rectus femoris* knee extensor muscles during stance showed an immediate positive effect on peak knee extension angle. In this study, NMES to both the *rectus femoris* and the *vastus lateralis* was the most effective at improving posture as the average peak knee extension angle during mid-stance increased by 2.9° in the left (more affected) limb and 6.5° in the right (less affected) limb from baseline exoskeleton walking without NMES (Table 1). Similar improvements were observed in total knee excursion by 5.2° in the left (more affected) limb and 7.0° in the right (less affected) limb from baseline exoskeleton walking. These improvements were observed despite a relatively simple FSM approach which activated NMES during stance phase, as indicated by time points at which the FSR under the foot was beyond a pre-set threshold. The slow gait velocity of this participant, who was assessed at the GMFCS III level, resulted in relatively large delays in both stimulation onset and offset compared to heelstrike and toe-off (Fig. 6). However, the flexibility of the controllable NMES design allows for the possibility of activating NMES during other phases of the gait cycle that may be beneficial for the target population, such as knee extension assistance in late swing. Therefore, future work should examine the optimal NMES duty cycle within the stride to improve knee extension and gait speed in individuals with crouch. Wearing the exoskeleton decreased gait speed, which is consistent with observations from previous studies [20]; after multiple practice sessions, gait speed improved and similar improvement may be possible in this participant, although that will be a focus of future study. Conversely, walking in the exoskeleton without assistance improved crouch compared to the prescribed AFO as measured by peak knee angle, an effect that may be attributed to its knee-ankle-foot architecture which restricts motion outside the sagittal plane. Further improvement in crouch was observed with the addition of NMES to the knee extensor muscles.

A similar, immediate orthotic effect on improved gait kinematics has been found in other studies when applying FES to the tibialis anterior [43], gastrocnemius [44], and quadriceps [45]. While the mean improvement seen in both limbs may have been significant only on the more affected limb in this case, these data are mainly included to demonstrate the NMES device operation and are too preliminary to draw any conclusions about the clinical effects given that these were from a limited number of strides in a single subject. Sufficient opportunity to practice is likely necessary to accommodate to these novel walking conditions before the full effect can be quantified, and will be done in multiple subsequent study visits. The improvement to the knee angle profile could likely be enhanced by increasing the intensity of stimulation delivered to the quadriceps muscles. Perhaps more importantly, we anticipate that improvements in muscle strength, spasticity, and unassisted gait kinematics could potentially be seen with long-term use which could further enhance the improvement in active knee extension.

NMES has been previously administered to lower limb muscles of children with cerebral palsy intermittently throughout the gait cycle in several studies, but the method of triggering the administration of electrical stimulation varied [29, 44, 46–48]. Some studies triggered electrical stimulation via a preset on:off stimulation time [44], while others initiated electrical stimulation with a remote controller operated by a physical therapist visually observing gait state transitions [46]. Other groups triggered the delivery of electrical stimulation via ipsilateral footswitches [32, 47, 48] from commercially-available, closed NMES digital systems. In the present study, electrical stimulation was triggered from our digitally-controlled NMES system using a finite state machine, based on foot-ground contact and knee angle [14], as the arbitrary digital input to the NMES. Accordingly, the NMES system herein described is suitable for future use with an exoskeleton. A combined exoskeleton NMES system provides significant advantages for precisely timed delivery of stimulation during walking. The exoskeleton architecture includes sensors that track the limb during walking which are not available in NMES-only systems. While the benefits of using motorized knee extension assistance from an exoskeleton [14, 15, 20] or FES [32] as treatments to improve crouch have been reported, few studies exist combining FES with exoskeleton assistance into a single, multi-modal treatment [33–39]. Moreover, while these published studies describe hybrid FES-exoskeletons that have been tested conceptually on healthy adults [33, 34] and used clinically for adults with spinal cord injury [35–38], to the best of our knowledge, no studies have outlined the electromechanical design of integrating a digitally-controlled NMES into a hybrid device in cerebral palsy, and no studies have presented experimental results on the application of a hybrid NMES exoskeleton in a pediatric population.

In the future, we propose that a system that combines powered exoskeleton knee extension assistance with NMES holds promising clinical significance. First, this system would provide a convenient opportunity for children with CP to incorporate NMES into their daily rehabilitation regiments, which could lead to additional improvements in functional movement outcomes as electrical stimulation has been shown to strengthen muscles in children with CP [49–51]. Further, the synchronization of this combined therapy could build a motor learning training paradigm to augment the effects of strengthening muscles. NMES administered in a synchronized manner could provide haptic biofeedback, which trains users to learn which muscles should be activated throughout specific phases of the gait cycle, and may lead to neuroplastic changes in the sensorimotor regions and associated pathways. Signaling muscle contraction with timed NMES, a rehabilitation technique described as providing “cutaneous cues” by Johnston et al., could train synchronized muscular contraction rhythms for walking and other cyclic motions in patients with cerebral palsy [52]. Electrical stimulation preferentially targets fast twitch muscle fibers, which may help increase the rate of force production, shown to be more impaired and more functionally relevant in CP than muscle weakness [53]. A final potential advantage of electrical stimulation to the quadriceps is to provide or increase the reciprocal inhibition of the spastic knee flexors [22]. More studies are needed to investigate the immediate and long-term effects of synchronized NMES administered to the quadriceps muscles and its efficacy as a treatment for crouch in children with cerebral palsy [32].

The novelty of the present study lies in both its initial clinical application to precisely trigger surface electrical stimulation to the quadriceps of a child with bilateral spastic CP, GMFCS Level III as well as the versatility of the digitally-controlled NMES interface that was designed. In previous studies that triggered the administration of electrical stimulation at certain gait events, such as heel strike, commercial gait training electrical stimulators were used that use only a footswitch as the trigger for stimulation [32, 47, 48]. This custom digitally-controlled NMES device offers the flexibility of triggering electrical stimulation to multiple muscles via an arbitrary digital input signal. Here we showed initial feasibility and tolerability of stimulating multiple knee extensor muscles that are synergistic with the intended robotic assistance from the exoskeleton. While our initial focus has been the knee, extensor muscles at the ankle, as well as the hip, also contribute to crouch [54] and these as well as other muscles, e.g. ankle dorsiflexors, are also excellent potential candidates for the use of our NMES system to improve gait in CP. Therefore, strengthening and/or assistance to those muscles [18] has and should be investigated further instead of or in combination with the knee extensors. Our controllable NMES device could easily be incorporated with robotic devices that target other joints for gait rehabilitation, such as the ankle exoskeleton for children with CP proposed by Lerner et al. [18], or otherwise could be used for a variety of therapeutic applications where precisely timed muscle activation assistance might be useful. The NMES device could also be programmed to stimulate muscles controlling different joints than those being assisted by the robotic exoskeleton. The ideal muscle targets for NMES and/ or joints for robotic assistance that produce the largest immediate and training effects will likely be patient-specific based on individual functional capabilities and treatment goals. Further, while not utilized in the present study, the digital control of the NMES calibration (amplitude, pulse width, and frequency) provides the means for modulating NMES waveforms automatically and in real-time, such as increasing NMES intensity as more assistance is needed.

Limitations in this proposed design and study include the simplicity of the FSM used for this experiment (two state FSM). It would be beneficial to test the robustness of NMES and exoskeleton assistance synchronization in more complex cases, such as a five-state FSM and adaptive controllers which we have recently developed for use with motorized assistance [42]. Testing the NMES-exoskeleton system on only one participant presents another limitation in making conclusions about its potential clinical effectiveness in other patients. Nevertheless, this study design aimed to investigate feasibility of integrating controllable NMES into an assistive device such as a robotic exoskeleton for gait rehabilitation by demonstrating its operation in one patient with the target gait pathology. We recognize that the clinical results presented, while interesting and encouraging, are preliminary and will require further study. In future work, it will be necessary to investigate the efficacy of combining NMES and exoskeleton powered assistance as a treatment for crouch gait, and comparing the improvements to crouch when using a combined treatment compared to each treatment in isolation, to better understand if the continued development of a combined NMES and exoskeleton treatment is viable and worthwhile for the long-term rehabilitation of crouch gait in children with CP and other neurological conditions. Finally, the lasting effects of these two interventions on gait function need to be evaluated by quantifying improvements in walking with device assistance after intensive training studies. However, since the effects of strength training in particular will not persist in the absence of regular training, either this device and/or progressive resistance training would likely be advocated to maintain or potentially enhance benefits.

## Conclusions

This report presents the design of a novel NMES system built around the architecture of a commercially-available four-channel, eight-electrode electrical muscle stimulator that can be digitally calibrated and triggered by an arbitrary external digital signal with low-latency (16.5 ± 13.5 ms) to administer electrical stimulation. The flexible control of the system allows this device to be incorporated into a variety of rehabilitation projects. The present study demonstrates the initial application of this system to trigger electrical stimulation to the quadriceps muscles of a child with cerebral palsy while walking in a pediatric robotic exoskeleton; this application resulted in immediate improvements to knee flexion during stance and should be investigated further as a treatment for children with crouch.

## Data Availability

The datasets used and/or analyzed during the current study are available from the corresponding author on reasonable request (damianod@cc.nih.gov).

## Abbreviations

CP: Cerebral palsy
GMFCS: Gross Motor Function Classification System
MAS: Modified Ashworth Scale
SCI: Spinal cord injury
NMES: Neuromuscular electrical stimulation
FES: Functional electrical stimulation
FSM: Finite state machine
VL: Vastus lateralis
RF: Rectus femoris
PCB: Printed circuit board
GUI: Graphical user interface
FDA: Food & Drug Administration
SPST: Single Pole Single Throw
NO: Normally-Open
IDE: Integrated Development Environment
LCD: Liquid crystal display
ROM: Range of Motion
AFO: Ankle-Foot Orthosis

## References

[1] Jensen A. Autologous cord blood therapy for infantile cerebral palsy: from bench to bedside. Obstet Gynecol Int. 2014;2014. Article ID 976321, 12 pages. 10.1155/2014/976321 PMC3956288 24695413

[2] Bax M, Goldstein M, Rosenbaum P, Leviton A, Paneth N, Dan B (2005). Proposed definition and classification of cerebral palsy, April 2005. Dev Med Child Neurol.

[3] Wren TA, Rethlefsen S, Kay RM (2005). Prevalence of specific gait abnormalities in children with cerebral palsy: influence of cerebral palsy subtype, age, and previous surgery. J Pediatr Orthop.

[4] Galey SA, Lerner ZF, Bulea TC, Zimbler S, Damiano DL (2017). Effectiveness of surgical and non-surgical management of crouch gait in cerebral palsy: a systematic review. Gait Posture.

[5] Rose J, Gamble JG, Burgos A, Medeiros J, Haskell WL (1990). Energy expenditure index of walking for normal children and for children with cerebral palsy. Dev Med Child Neurol.

[6] Bottos M, Gericke C (2003). Ambulatory capacity in cerebral palsy: prognostic criteria and consequences for intervention. Dev Med Child Neurol.

[7] Sutherland D, Santi M, Abel M (1990). Treatment of stiff-knee gait in cerebral palsy: a comparison by gait analysis of distal rectus femoris transfer versus proximal rectus release. J Pediatr Orthop.

[8] Rodda J, Graham HK, Nattrass G, Galea MP, Baker R, Wolfe R (2006). Correction of severe crouch gait in patients with spastic diplegia with use of multilevel orthopaedic surgery. JBJS.

[9] Thompson N, Baker R, Cosgrove A, Corry I, Graham H (1998). Musculoskeletal modelling in determining the effect of botulinum toxin on the hamstrings of patients with crouch gait. Dev Med Child Neurol.

[10] Damiano DL, Alter KE, Chambers H (2009). New clinical and research trends in lower extremity management for ambulatory children with cerebral palsy. Phys Med Rehabil Clin.

[11] Damiano DL, Arnold AS, Steele KM, Delp SL (2010). Can strength training predictably improve gait kinematics? A pilot study on the effects of hip and knee extensor strengthening on lower-extremity alignment in cerebral palsy. Phys Ther.

[12] Rethlefsen SA, Yasmeh S, Wren TA, Kay RM (2013). Repeat hamstring lengthening for crouch gait in children with cerebral palsy. J Pediatr Orthop.

[13] Dreher T, Vegvari D, Wolf SI, Geisbüsch A, Gantz S, Wenz W (2012). Development of knee function after hamstring lengthening as a part of multilevel surgery in children with spastic diplegia: a long-term outcome study. JBJS.

[14] Lerner ZF, Damiano DL, Park H-S, Gravunder AJ, Bulea TC (2016). A robotic exoskeleton for treatment of crouch gait in children with cerebral palsy: design and initial application. IEEE Trans Neural Syst Rehabil Eng.

[15] Lerner ZF, Damiano DL, Bulea TC. A robotic exoskeleton to treat crouch gait from cerebral palsy: Initial kinematic and neuromuscular evaluation. In: 2016 38th Annual International Conference of the IEEE Engineering in Medicine and Biology Society (EMBC). Orlando: IEEE; 2016.. 10.1109/EMBC.2016.7591169 28324959

[16] Banala SK, Kim SH, Agrawal SK, Scholz JP. Robot assisted gait training with active leg exoskeleton (ALEX). In: 2008 2nd IEEE RAS & EMBS International Conference on Biomedical Robotics and Biomechatronics. Scottsdale: IEEE; 2008.

[17] Lerner ZF, Gasparri GM, Bair MO, Lawson JL, Luque J, Harvey TA (2018). An untethered ankle exoskeleton improves walking economy in a pilot study of individuals with cerebral palsy. IEEE Trans Neural Syst Rehabil Eng.

[18] Lerner ZF, Harvey TA, Lawson JL (2019). A battery-powered ankle exoskeleton improves gait mechanics in a feasibility study of individuals with cerebral palsy. Ann Biomed Eng.

[19] Bayon C, Ramírez O, Serrano JI, Del Castillo M, Pérez-Somarriba A, Belda-Lois JM (2017). Development and evaluation of a novel robotic platform for gait rehabilitation in patients with cerebral palsy: CPWalker. Robot Auton Syst.

[20] Lerner ZF, Damiano DL, Bulea TC (2017). A lower-extremity exoskeleton improves knee extension in children with crouch gait from cerebral palsy. Sci Transl Med.

[21] Damiano DL, Prosser LA, Curatalo LA, Alter KE (2013). Muscle plasticity and ankle control after repetitive use of a functional electrical stimulation device for foot drop in cerebral palsy. Neurorehabil Neural Repair.

[22] Bosques G, Martin R, McGee L, Sadowsky C (2016). Does therapeutic electrical stimulation improve function in children with disabilities? A comprehensive literature review. J Pediatr Rehabil Med.

[23] Kang B-S, Bang MS, Jung SH (2007). Effects of botulinum toxin a therapy with electrical stimulation on spastic calf muscles in children with cerebral palsy. Am J Phys Med Rehabil.

[24] Mooney JA, Rose J (2019). A scoping review of neuromuscular electrical stimulation to improve gait in cerebral palsy: the arc of Progress and future strategies. Front Neurol.

[25] Field-Fote EC (2004). Electrical stimulation modifies spinal and cortical neural circuitry. Exerc Sport Sci Rev.

[26] Damiano DL, Abel MF (1998). Functional outcomes of strength training in spastic cerebral palsy. Arch Phys Med Rehabil.

[27] Currier D, Mann R (1983). Muscular strength development by electrical stimulation in healthy individuals. Phys Ther.

[28] Peckham PH, Knutson JS (2005). Functional electrical stimulation for neuromuscular applications. Annu Rev Biomed Eng.

[29] Seifart A, Unger M, Burger M (2009). The effect of lower limb functional electrical stimulation on gait of children with cerebral palsy. Pediatr Phys Ther.

[30] Kottink AI, Oostendorp LJ, Buurke JH, Nene AV, Hermens HJ, IJzerman MJ (2004). The orthotic effect of functional electrical stimulation on the improvement of walking in stroke patients with a dropped foot: a systematic review. Artif Organs.

[31] Laufer Y, Hausdorff JM, Ring H (2009). Effects of a foot drop neuroprosthesis on functional abilities, social participation, and gait velocity. Am J Phys Med Rehabil.

[32] Khamis S, Martikaro R, Wientroub S, Hemo Y, Hayek S (2015). A functional electrical stimulation system improves knee control in crouch gait. J Child Orthop.

[33] Del-Ama AJ, Gil-Agudo Á, Pons JL, Moreno JC (2014). Hybrid FES-robot cooperative control of ambulatory gait rehabilitation exoskeleton. J Neuroeng Rehabil.

[34] Kirsch NA, Bao X, Alibeji NA, Dicianno BE, Sharma N (2018). Model-based dynamic control allocation in a hybrid Neuroprosthesis. IEEE Trans Neural Syst Rehabil Eng.

[35] Ha KH, Quintero HA, Farris RJ, Goldfarb M. Enhancing stance phase propulsion during level walking by combining FES with a powered exoskeleton for persons with paraplegia. In: 2012 Annual International Conference of the IEEE Engineering in Medicine and Biology Society: IEEE; 2012. 10.1109/EMBC.2012.6345939 PMC3694438 23365900

[36] Ha KH, Murray SA, Goldfarb M (2015). An approach for the cooperative control of FES with a powered exoskeleton during level walking for persons with paraplegia. IEEE Trans Neural Syst Rehabil Eng.

[37] Bulea TC, Kobetic R, Audu ML, Triolo RJ (2013). Stance controlled knee flexion improves stimulation driven walking after spinal cord injury. J Neuroeng Rehabil.

[38] Del-Ama AJ, Koutsou AD, Moreno JC, De-Los-Reyes A, Gil-Agudo Á, Pons JL. Review of hybrid exoskeletons to restore gait following spinal cord injury. J Rehabil Res Dev. 2012;49(4):497–514. 10.1682/jrrd.2011.03.0043 22773254

[39] Chang SR, Nandor MJ, Li L, Kobetic R, Foglyano KM, Schnellenberger JR (2017). A muscle-driven approach to restore stepping with an exoskeleton for individuals with paraplegia. J Neuroeng Rehabil.

[40] Maurer DD, Sorenson PD. Electrical stimulation system: Google Patents; 1977.

[41] Thompson DL, Roline GM, Harrington RJ. Digital circuit for control of gradual turn-on of electrical tissue stimulators: Google Patents; 1985.

[42] Chen J, Hochstein J, Kim C, Damiano D, Bulea T. Design advancements toward a wearable pediatric robotic knee exoskeleton for overground gait rehabilitation. In: 2018 7th IEEE International Conference on Biomedical Robotics and Biomechatronics (Biorob). Enschede: IEEE; 2018. 10.1109/biorob.2018.8487195 PMC10436700 37600973

[43] Pierce SR, Orlin MN, Lauer RT, Johnston TE, Smith BT, McCarthy JJ (2004). Comparison of percutaneous and surface functional electrical stimulation during gait in a child with hemiplegic cerebral palsy. Am J Phys Med Rehabil.

[44] Comeaux P, Patterson N, Rubin M, Meiner R (1997). Effect of neuromuscular electrical stimulation during gait in children with cerebral palsy. Pediatr Phys Ther.

[45] Postans NJ, Granat MH (2005). Effect of functional electrical stimulation, applied during walking, on gait in spastic cerebral palsy. Dev Med Child Neurol.

[46] Carmick J (1993). Clinical use of neuromuscular electrical stimulation for children with cerebral palsy, part 1: lower extremity. Phys Ther.

[47] Durham S, Eve L, Stevens C, Ewins D (2004). Effect of functional electrical stimulation on asymmetries in gait of children with hemiplegic cerebral palsy. Physiotherapy.

[48] Pierce SR, Laughton CA, Smith BT, Orlin MN, Johnston TE, McCarthy JJ (2004). Direct effect of percutaneous electric stimulation during gait in children with hemiplegic cerebral palsy: a report of 2 cases. Arch Phys Med Rehabil.

[49] Kerr C, McDowell B, Cosgrove A, Walsh D, Bradbury I, McDonough S (2006). Electrical stimulation in cerebral palsy: a randomized controlled trial. Dev Med Child Neurol.

[50] Carmick J (1995). Managing equinus in children with cerebral palsy: electrical stimulation to strengthen the triceps surae muscle. Dev Med Child Neurol.

[51] Stackhouse SK, Binder-Macleod SA, Stackhouse CA, McCarthy JJ, Prosser LA, Lee SC (2007). Neuromuscular electrical stimulation versus volitional isometric strength training in children with spastic diplegic cerebral palsy: a preliminary study. Neurorehabil Neural Repair.

[52] Johnston TE, Prosser LA, Lee SC (2008). Differences in pedal forces during recumbent cycling in adolescents with and without cerebral palsy. Clin Biomech.

[53] Moreau NG, Falvo MJ, Damiano DL (2012). Rapid force generation is impaired in cerebral palsy and is related to decreased muscle size and functional mobility. Gait Posture.

[54] Rodda J, Graham H. Classification of gait patterns in spastic hemiplegia and spastic diplegia: a basis for a management algorithm. Eur J Neurol. 2001;8:98–108. 10.1046/j.1468-1331.2001.00042.x 11851738

